# Mycelium-Based Composites: Surveying Their Acceptance by Professional Architects

**DOI:** 10.3390/biomimetics9060333

**Published:** 2024-05-30

**Authors:** Anna Lewandowska, Agata Bonenberg, Maciej Sydor

**Affiliations:** 1 Institute of Interior Design and Industrial Design, Faculty of Architecture, Poznan University of Technology, 61-131 Poznań, Poland; lewaandowsk.anna@gmail.com (A.L.); agata.bonenberg@put.poznan.pl (A.B.); 2 Department of Woodworking and Fundamentals of Machine Design, Faculty of Forestry and Wood Technology, Poznań University of Life Sciences, 60-637 Poznań, Poland

**Keywords:** fungi, biomaterials, architect perception, user acceptance, willingness to adopt, aesthetic preferences, survey research, small-scale prototypes

## Abstract

Mycelium-based composites (MBCs) are biomaterials with scientifically proven potential to improve sustainability in construction. Although mycelium-based products are not entirely new, their use in engineering presents challenges due to the inherent properties of this fungal material. This study investigated professional architects’ and interior designers’ perceptions of MBCs, focusing on familiarity, aesthetic appeal, and willingness to use. The first phase of the survey explored respondents’ views on material-related ecological design principles. In the second phase, respondents evaluated ten small architectural objects crafted from MBCs, focusing on form, detail, and visual appeal. The last phase of the survey measured their interest in using mycelium in their design work. The results revealed that MBCs were relatively unknown among the surveyed professionals; only every second respondent knew this material. Despite this, 90% found MBCs visually appealing after seeing the examples. Interestingly, the natural, unprocessed appearance of the material was assessed as less aesthetically pleasing, with thermal treatment improving its perceived value. Architects were more receptive to using MBCs in their professional projects for customers than for personal use. This observation points to a ‘double standard’: professional architects are more open to using MBCs in projects not intended for their own use.

## 1. Introduction

Architects constantly search for innovative, more sustainable materials to address the challenges of modern civilization. Due to population growth and technological advancements, global waste generation is projected to increase from 2.01 billion tons in 2016 to 3.40 billion tons by 2050 [1]. Construction and demolition waste (CDW) presents difficulties in effective and accurate administration [2]. Additionally, the construction sector is a significant driver of climate change, responsible for 38% of global energy-related CO_2_ emissions (according to the UN’s 2020 ‘Global Status Report for Buildings and Construction’ [3]). These figures highlight the limitations of current building materials in terms of sustainability.

A promising class of architectural materials is mycelium-based composites (MBCs). By 2022, research had yielded at least 92 original scientific papers related to MBCs [4]. Its potential applications have been explored in scientific publications, covering aspects such as manufacturability [5], low cost [6], electronic applications [7], architectural [8] and green building applications [9], furniture and art use [10], and sustainability benefits [11]. Research has demonstrated that combining mycelium biofabrication with a permanent knitted textile formwork significantly enhanced the mechanical performance of the resulting mycelium-textile biocomposites. This advancement paves the way for their utilization in large-scale construction components [12,13], because developing mycelium composites with a hierarchical porous structure has yielded materials with good thermal and mechanical properties [14]. These composites exhibit high porosity, low thermal conductivity, and good energy absorption, positioning them as sustainable alternatives to lightweight plastic foams commonly used in construction [14]. Additionally, the electrical sensitivity of mycelium-bound composites to variations in moisture content presents novel possibilities for creating active building components [15].

MBCs hold weighty promise for sustainable architecture because they utilize byproducts from other industries [11]. MBCs are fully biodegradable, minimizing their environmental impact [16]. Their production requires less energy than conventional building materials, and MBCs boast a significantly lower carbon footprint in the construction sector [17]. Due to their compostability, used MBC products can be repurposed as agricultural fertilizer. At the end of their lifespan, MBC products can seamlessly decompose and integrate into the natural environment [18]. MBCs have favorable thermal and sound insulation properties; they are non-flammable.

The synergy of these unique characteristics underscores the versatility and innovative potential of mycelium-based materials (MBCs) within the construction industry. The range of applications, from lightweight semi-structural building components to high-performance insulation materials and aesthetically pleasing interior design elements, positions MBCs as promising solutions for architects and interior designers seeking sustainable alternatives in their projects.

Mycelium-based composites (MBCs) have characteristics that position them as biomimetic materials for architecture. The material’s functionality is inspired by nature, and nature’s design is mimicked in the end products. Mycelium naturally grows into desired shapes, potentially reducing the need for traditional processing and construction techniques. This mimics natural self-assembly processes seen in biological structures. Mycelium responds to environmental stimuli by adjusting its growth patterns. This mimics the ability of natural materials to adapt to their surroundings, potentially leading to building components that regulate temperature or humidity. Mycelium, the root-like structure of fungi, forms intricate networks that optimize strength. Similar natural lightweight structures are found in bones and plants. Mycelium composites utilize natural materials like consumer, industrial, and agricultural waste as growth media, mimicking natural composite structures found in bone, wood, and other lignocellulosic materials.

Although mycelium is becoming increasingly popular in architecture, this biomimetic material is not used on a large scale because it still poses many technological challenges [12]. The main problem is that its strength properties during production have not yet been fully stabilized [19]. However, the development of MBCs is accelerating, as evidenced by the rapidly increasing number of patents related to mycelium-based materials technology since 2007 [20].

Using fungi in MBCs raises concerns about potential health effects [18], but they are likely to be a safer option than particleboard or fiberboard, which often contain formaldehyde and other potentially harmful chemicals. Overcoming public perceptions of fungi as inherently harmful presents a challenge [21].

The inherent variability in the visual characteristics of MBCs presents a challenge for architectural applications. As living materials, mycelium-based products exhibit non-uniform textures on surfaces, varying depending on processing methods. Additionally, they may exhibit visual changes over time. Concerns about fungi and the material’s specific appearance justify investigating this material’s acceptability in design applications. A study by Bonenberg et al. (2019) explored public perceptions through a survey that measured reactions to small, decorative interior design products made of MBCs. It is important to note that the respondents were architecture students evaluating furniture and appliance-like objects. The cited study found that while MBCs can be perceived as fascinating and sustainable, their fungal origin and unusual appearance raise some concerns [22].

Selecting sustainable materials is just the first step. Widespread adoption and proper implementation by qualified professionals are critical for realizing truly eco-friendly buildings. This study aims to expand the findings on small interior design products made of MBCs and examine the acceptance of this material among practicing professional architects and interior designers in architectural design.

## 2. Materials and Methods

### 2.1. Respondents Involved in the Study

The survey included 50 participants, ensuring diverse perspectives within professional architecture and interior design. This diversity encompassed factors such as age (19–55 years old), gender (25 women and 25 men), and professional roles (architects, interior designers, assistant architects). All participants held degrees in either architecture or interior design, demonstrating their foundational knowledge and expertise in the field. Their professional experience further strengthened their qualifications. Notably, 84% of the participants fell within the 19–35 age range, positioning them as a group likely to be at the forefront of shaping future trends in architecture and interior design. For transparency, it is important to clarify that all participants in this survey were recruited from Poland. As a result, the respondents mainly came from Poland (47 people), but there were two people from Ukraine and one from Greece. In this study sample, 44 people were employed in Poland, and 6 people worked in other countries, i.e., 2 people in the Netherlands, 3 in Denmark, and 1 person in Great Britain.

### 2.2. Examples Used in the Survey and Their Selection Criteria

Table 1 lists these small-scale architectural objects used in the study.

The small-scale architectural objects used in this study were chosen based on three criteria: their purpose, their scale concerning the intended use of mycelium-based composites (MBCs), and the specific method of using the MBC within the object. The objects shown in Table 1 had the following characteristics:

- These objects served a dual purpose: exhibition and experimentation. Created for presentation at various fairs, exhibitions, and festivals, they showcased the potential of MBCs for use in building structures for a broad audience.
- They were objects from the realm of small-scale architecture. (It is worth mentioning that there is no universally accepted definition of small-scale architecture. It generally refers to architectural creations smaller than landscapes, infrastructure, or buildings, including street furniture, public art installations, landscape features and objects, and indoor objects).
- The objects used MBCs to fill or create their structures.

These objects have been widely described and analyzed in the scientific literature (Myco Tree [33], Circular Garden [5,8], Grooving Pavilion [34], Mycotectural Alpha [35], Hy-Fi [36], Shell Mycelium Pavilion [8], My-co Space [37], El Monolito Micelio [13], Hayes Pavilion [38], and Mycelium Textile Pavilion [39]).

### 2.3. The Survey Design and Data Collection Method

The survey questionnaire was divided into three sections:

- Section 1. Knowledge about biomaterials, including MBC (eco-friendliness, utilization of biomaterials). The first two questions were closed-ended (answers: yes or no), and the third question was open-ended, aimed at exploring the respondents’ awareness of biomaterials, the popularity of biomaterial utilization among respondents, and determining the most commonly used biodegradable materials in architectural projects. Questions 1.4 and 1.5 were closed-ended (answers: yes or no) and were aimed at exploring the respondents’ awareness of the possibilities of using MBCs.
- Section 2. Evaluation of the aesthetic level of objects (form, detail, visual perception). The three questions in this section aimed to obtain respondents’ assessments of three aesthetics-related categories: form, detail, and overall visual perception. Respondents provided ratings on a five-point Likert scale, one of the fundamental and most commonly used psychometric tools in educational and social science research [40].
- Section 3. Assessment of material acceptance level and personal impressions. Following their analysis of the photographic examples in Section 2 of the survey, respondents were asked to share their opinions on MBC. This part of the survey gauged respondents’ perception of mycelium-based composites (MBCs) in terms of aesthetics (visual appeal and harmony), design potential (shaping small architectural forms), and their interest in incorporating MBC into both professional and personal design projects. All five questions in this section employed a five-point Likert scale for responses.

A complete list of the survey questions used in this study is provided in Appendix A for reference. The questions are also cited throughout Section 3.

The survey was conducted anonymously. No direct identifiers were collected. We collected three indirect identifiers: gender, age, and professional role of the study participant, meaning that the opinions provided about the tested material could not be linked to any individual participant. After being informed about the purpose and scope of the questions, all participants freely consented to participate in the study and provided informed consent before commencing their responses. The survey was administered remotely to ensure unbiased responses, minimizing external influence; consequently, the study relied on photographs of the objects listed in Table 1. The photographs were methodically chosen to facilitate a comprehensive understanding of the objects. The first image presented a complete view of each object, while subsequent images zoomed in to capture the finer details, such as texture or craftsmanship. All photographs depicted the objects in natural daylight without any artificial filters, ensuring an accurate representation.

## 3. Results and Discussion

### 3.1. Results of Section 1. Knowledge about Biomaterials, Including MBCs

Questions 1.1 and 1.2 pertained to the declared and actual impact of ecological requirements on architectural design:

- Question 1.1. Do you believe ecology plays a significant role in shaping contemporary architecture?
- Question 1.2. Do you incorporate biodegradable materials in your architectural or interior design projects?

**Figure 1:**
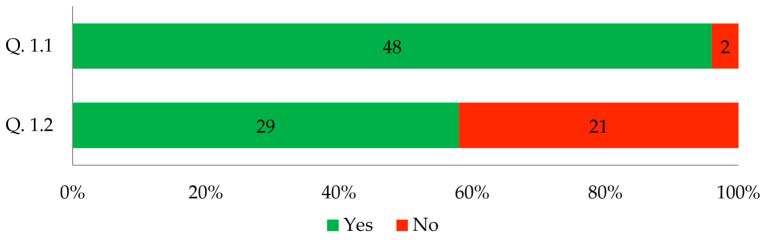
Respondents’ answers to questions 1.1 and 1.2 (*n* = 50).

Figure 1 illustrates the answers to these questions.

The answers to questions 1.1 and 1.2 show that although almost all architects believed that ecology has an impact on shaping contemporary architecture, only 58% used biodegradable materials in their own architectural or interior design projects. This result indicates that the requirement for eco-friendliness has had less impact on the materials used in contemporary architecture than the surveyed architects themselves thought. Probably, there is a low knowledge of sustainable building materials among architects, as revealed by a qualitative study conducted by Umar et al. [41]. Thomsen and Tamke argued that establishing a bio-based material paradigm in architecture can promote sustainable building practices [42]. Yohe discussed the need for architecture to consider the social body and diversity, emphasizing the importance of material properties such as elasticity in promoting inclusivity [43]. While there is growing interest in sustainable and ecological materials in architecture, the level of their intensive use by architects is unsatisfactory. This may be related to the low budgets of investors who strive to limit project expenses. Often, the cheapest material solutions are not eco-friendly. While architects understand the ecological demands of contemporary architecture and the need for their projects to meet them, budget constraints [6] and limited knowledge of biomaterials restrict their available options [8,9,39,41].

In response to open-ended question 1.3, “If yes, what specific biodegradable materials have you used in your designs?” the following responses were collected: straw, reed, wood, recycled wood, fabric from pineapple fibers, rammed earth, bioplastic from mixed algae, branches, stone, bio-fillings for 3D printers, bamboo, reclaimed stone, shells, straw bale, bamboo, hemp, flax, potato starch, grains, sugarcane, flowers. A total of 22 materials were indicated. The responses “wood” and “mostly wood” were repeated 5 times. Wood was the biodegradable material most commonly used by the surveyed architects.

These findings are in line with those of other studies. Wood is a commonly used biodegradable material in architectural projects [44]. It is highlighted in numerous articles as a significant material in the construction industry [45]. Wood-based construction materials, including chipboard, plywood and laminates, have been widely studied for their biodegradability and environmental quality, showing that wood-based materials are aerobically and anaerobically biodegradable [2]. Additionally, the weathering resistance of bio-based facade materials, including natural wood, has been investigated, emphasizing the need for optimizing the appearance of wood facades in outdoor conditions [46]. The activity of living organisms on wood as a construction material has also been discussed, highlighting the impact of biodeterioration on the structural performance of wood [47]. Therefore, wood is a commonly used biodegradable material in architectural projects, with its properties and potential for sustainable construction having been studied extensively.

The closing questions of this section examined knowledge about MBCs. The questions were as follows:

- Question 1.4. Are you familiar with mycelium-based composites (MBCs) as a biomaterial?
- Question 1.5. Have you heard of using any MBC as a building material?

**Figure 2:**
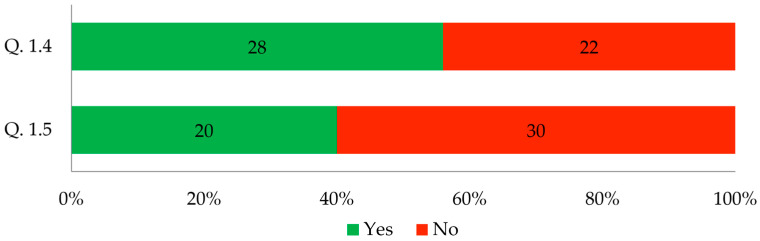
Respondents’ answers to questions 1.4 and 1.5 (*n* = 50).

The responses to these questions are compiled in Figure 2.

The results shown in Figure 2 indicate that MBCs are not widely known biomaterials among architects and interior designers. In this study, 56% of the respondents had heard about using mycelium as a biocomposite matrix. This was just over half of the respondents. However, only 40% of the surveyed architects had heard about using MBC as a building material, which contradicts the literature reports. In the book *Fungal Architectures* [43], a reprint of the Special Issue published in *Biomimetics*, the authors mention that the cultivation, preparation, and exploitation of mycelium composites are of interest to architects, among other professionals.

These materials are still in the testing phase, and a few examples of MBCs have been used as building materials. Their use mainly revolves around the creation of structures of small architectural forms. Popularizing biomaterials among architects can increase their use in construction, perhaps in new forms and ideas. Almpani-Lekka et al. [34] and Ghazvinian and Gursoy [48] findings support this claim. These authors noted that architects have recently designed and constructed various experimental projects using mycelium-based composites (MBCs). This suggests that MBCs attract interest from architects, but their full-scale integration into architecture has yet to be realized.

### 3.2. Results of Section 2: Assessment of the Aesthetics of Selected Architectural Objects

**Figure 3:**
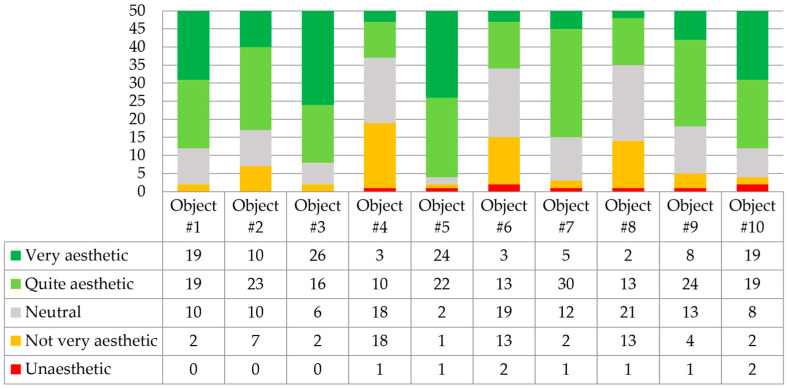
Respondents’ answers to question 2.1 (*n* = 50).

The questions in the second section of the survey assessed the design quality of 10 small-scale architectural realizations. Question 2.1 was formulated: “Please evaluate the overall form of the small-scale architectural objects”. The answers are shown in Figure 3.

The most aesthetically pleasing to the respondents were the largest and most complex architectural forms. Project number 5 (Hy-Fi) was rated the highest in aesthetics, followed closely by the aesthetics of project number 3 (The Growing Pavilion) and the intricate internal form of Myco Tree, project number 1. The least aesthetic was considered to be the form of project number 4 (Mycotectural Alpha), followed by projects 6 (Shell Mycelium Pavilion) and 8 (Monolito Mycelio). It is worth emphasizing that large-scale objects are challenges due to insufficient (but still improving [48]) material strength. Dessi-Olive presents two fabrication strategies for growing large building units; these strategies are based on myco-welded slabs [13]. Large-scale structural design applications lack diversity beyond brick/block, monolithic casting, or 3D printing-based approaches [49].

**Figure 4:**
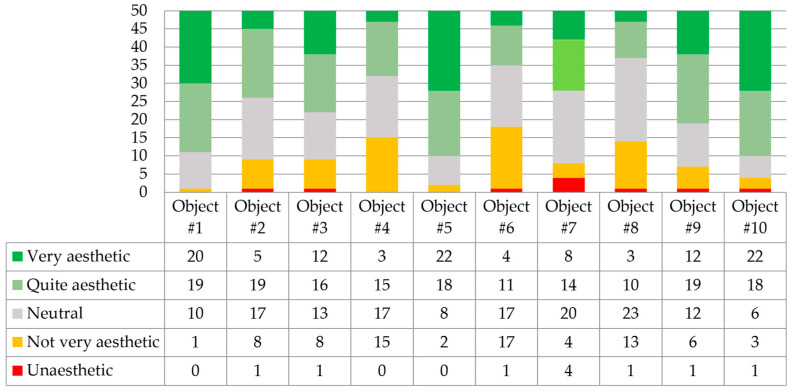
Respondents’ answers to question 2.2 (*n* = 50).

Question 2.2 was, “To what extent do you find the architectural details of these objects to be well executed and precise?”. The answers are shown in Figure 4.

Respondents recognized project number 5, HyFi Tower, as the project with the highest quality and precision of architectural detail execution. The execution quality of the Myco Tree project was also highly rated. Project number 6, Shell Mycelium Pavilion, was considered the least aesthetic in detail quality. Low ratings were also given to Monolito Mycelio in this category.

**Figure 5:**
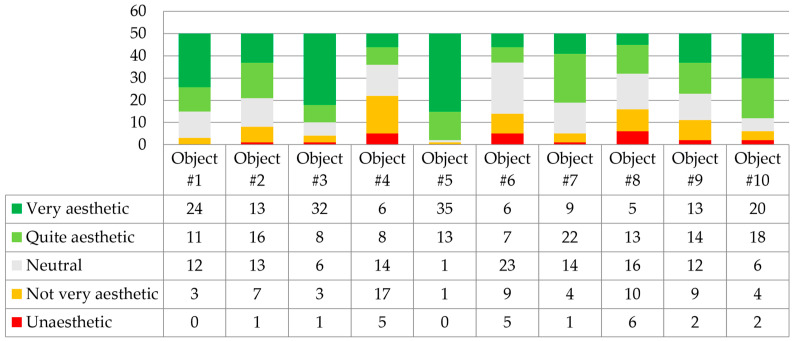
Respondents’ answers to question 2.3 (*n* = 50).

Question 2.2: “In your opinion, do the designs exhibit a high degree of visual interest?”. The answers are shown in Figure 5.

Respondents considered project number 5, once again Hy-Fi by David Benjamin and The Living Architects, as the most visually interesting. Project number 3, The Growing Pavilion by Company New Heroes, was also highly rated. Respondents indicated that the least visually interesting projects were project number 4, Mycotectural Alpha, and project number 6, Shell Mycelium Pavilion.

Comparing the above results, two correlations were observed. The projects deemed least visually interesting were structures with low-quality execution of detail and uninteresting form.

Interestingly, Mycotectural Alpha and Shell Mycelium Pavilion, considered the least aesthetic, are implementations in which the mycelium was not subjected to thermal processing. Therefore, it can be concluded that naturally developing mycelium with visible biological structures on the surface may be deemed aesthetically unappealing for architectural applications.

### 3.3. Results of Section 3: Evaluation of the Aesthetics of MBCs

The aesthetic appeal of projects using mycelium-based composites was rated on a five-point scale based on three questions.

- Question 3.1. How visually attractive do you find mycelium-based composites (MBCs)?
- Question 3.2. How visually pleasant or harmonious do you find MBCs?
- Question 3.3. In your opinion, do MBCs offer creative possibilities for shaping small architectural forms?

**Figure 6:**
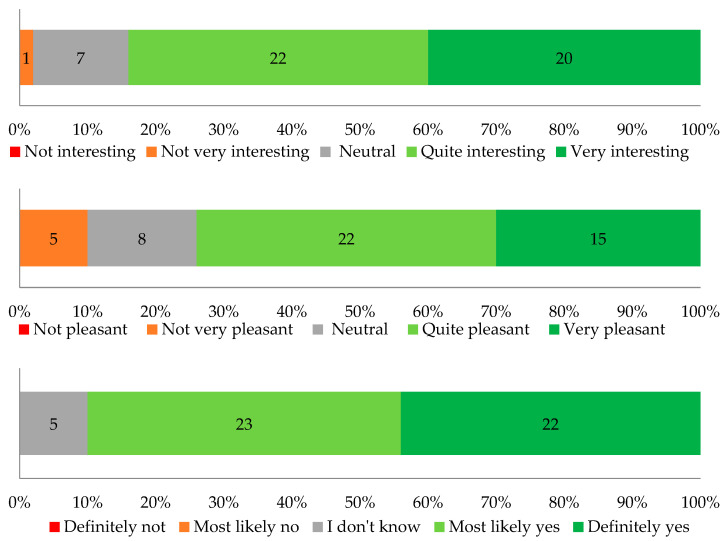
Comparative results of questions 3.1, 3.2, and 3.3 using a 5-point rating scale.

Figure 6 shows the results of this evaluation. 98% of respondents found MBCs visually attractive, while 90% considered them visually pleasant or harmonious. Comparing these results with the responses to questions 1.4 (“Are you familiar with mycelium-based composites (MBCs) as a biomaterial?”) and 1.5 (“Have you heard of using MBCs as a building material?”), where respondents showed limited knowledge of MBC, one can conclude that popularizing this biomaterial among architects may expand its utilization on a larger scale, perhaps in new forms.

It is worth mentioning that the literature emphasizes that sustainable and natural-looking building materials are perceived as more aesthetic. Natural building materials are perceived as more eco-friendly [50].

MBCs may raise concerns; therefore, the third section of the survey concluded with two crucial questions:

- Question 3.4. Would you consider MBCs in your professional design work?
- Question 3.5. Would you consider MBCs in a design project for your personal use?

**Figure 7:**
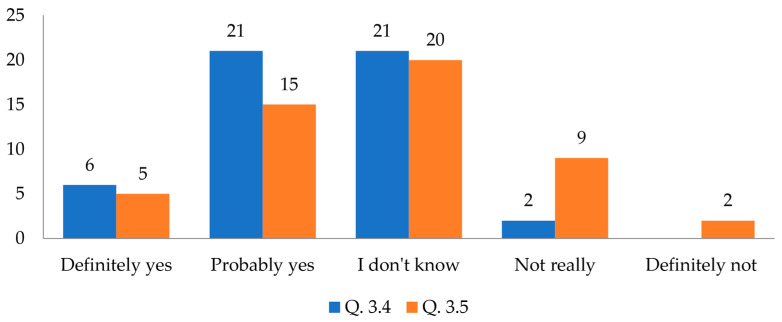
Comparative results of questions 3.4 and 3.5 using a 5-point rating scale.

Figure 7 highlights the disparity in responses depending on whether MBCs would be used in a professional project or for personal use. MBCs would be willingly utilized by architects in their projects but less willingly in their projects for their own use. This points to double standards but also indicates personal concerns related to the specific nature of the material. These concerns may involve the sensory properties of the material. Since respondents evaluated projects based on photographs, they may have worried that the material could be unpleasant to touch or give off unpleasant odors.

These results align with our previous findings from a study on architecture students, who represent individuals involved in interior design and possess competence in the architectural field. Similar to the current study, MBCs generally received positive or neutral evaluations. However, objects made from ceramic reference material were ultimately preferred. While the ecological benefits of MBCs were acknowledged, respondents hesitated to use them in their homes [22].

The survey revealed that many respondents were unfamiliar with MBCs in architecture. Having never encountered a built example, they lacked awareness of the material’s ecological benefits, availability, or cost. This knowledge gap might have influenced their responses to question 3.5, as they may have perceived MBCs as expensive or difficult to obtain.

While using living organisms like fungi might raise safety concerns due to associations with mold, educating architects about biomaterials like MBCs is crucial to address these concerns and explore their potential.

It is worth pointing out the study’s limitations:

- The results may primarily reflect the preferences of young professionals in a European geographic region and with an architectural educational background.
- Studying the acceptance level of a material in the implementation phase is challenging. Due to the subjectivity of respondents’ aesthetic sense, it is difficult to determine the most aesthetic and acceptable forms of material application.
- Interestingly, the respondents’ evaluation primarily focused on the aesthetic appeal of the MBC examples. While visual aspects are important, it is crucial to acknowledge that only half of the participants were familiar with MBCs. This limited awareness of the materials’ properties and processing methods might have influenced their evaluation.

## 4. Conclusions

The study investigated a cohort of 50 participants (25 women and 25 men) aged 19–55, all with degrees in architecture or interior design, with a high concentration under 35 years old. The research employed a mix of closed-ended yes/no, open-ended, and one-point consumer test questions using five-point scales. Based on the findings, the following conclusions were drawn:

- Mycelium-based composites (MBCs) are a relatively unknown material among surveyed architects. Only 56% of respondents had heard about using this biomaterial as a decorative material, and less than half had heard about using MBC as a structural or semi-structural material.
- Popularizing this biomimetic material among architects could lead to its more comprehensive application, perhaps in new forms and ideas for its utilization, as after analyzing examples, 90% of respondents found the material visually appealing. As the literature review showed, MBC emerges as a viable, circular, and ecologically responsible alternative to materials currently used in architecture. However, the visual aspect plays a significant role in the choice of materials for architects and may be a crucial aspect for successful product adaptation within the design community. The survey results show a positive visual response to MBC; 90% of respondents found the material visually appealing. The least aesthetically pleasing projects were those in which MBC was not subjected to thermal processing. The most aesthetic ones were those in which MBC underwent such processing. Therefore, the conclusion arises that naturally developing mycelium, with visible organisms on the surface, may be deemed unaesthetic in projects.
- Despite 96% of respondents believing ecology influenced contemporary architecture, only 58% used biodegradable materials in their projects. These results suggest that ecology has had a more negligible impact on the materials used in contemporary architecture than architects perceive.
- MBC would be willingly utilized by architects in their professional projects but less willingly in projects for their own use. This points to double standards but also indicates personal concerns related to the specific nature of the material.

Composite architectural materials based on mycelium are beneficial from an ecological point of view but not entirely accepted by professionals. However, this acceptance may increase with the popularization of the material and education about its environmental benefits and unique aesthetic qualities. Considering the cited literature and the results presented here, we can expect an increase in acceptance of MBCs among architects as familiarity with the materials and their benefits grows. However, MBCs possess a distinct aesthetic that may not universally appeal. Nevertheless, they must find a loyal user base among architects who value sustainable design solutions.

## Figures and Tables

**Table 1:** Small-scale architecture items used in the survey.

No.	Information	Photography	No.	Information	Photography
1	The Myco Tree, 2017 by Dirk E. Hebel and Philippe Block (photo from [23])	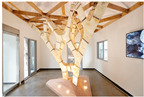	6	The Shell Mycelium Pavilion, 2016 by Beetles 3.3 and Yassin Areddia Designs (photo from [24])	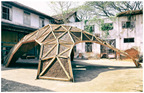
2	The Circular Garden, 2019 by Carlo Ratti (photo Marco Beck Peccoz), literature source [25]	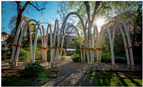	7	The My-co Space, 2021 (photo by Carlina Teteris), literature source [26]	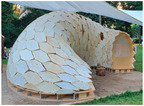
3	The Growing Pavilion, 2019 by Pascal Leboucq, Lucas De Man, and Eric Klarenbeek (photo Eric Melander), literature source [27]	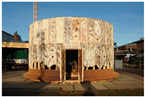	8	El Monolito Micelio, 2018 by Jonathan Dessi-Olive (photo by J. D.-O.), literature source [28]	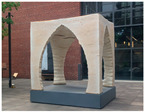
4	The Mycotectural Alpha, 2009 by Philip Ross (photo from [29])	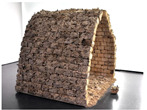	9	The Hayes Pavilion, 2023 by Simon Carroll (photo from [30])	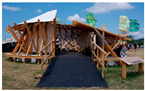
5	The Hy-Fi, 2014 by David Benjamin (photo by Kris Graves), literature source [31]	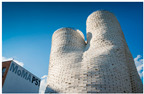	10	The Mycelium Textile Pavilion, 2022 by Nikolaj Emil Svenningsen, Sean Lyon, Søs Christine Hejselbæk (photo from [32])	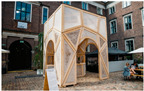

## Data Availability

All data obtained during the survey research and subsequent analysis are included in this article.

## Appendix A

Section 1. The first section of the survey consisted of general questions regarding ecology in architecture, the use of biomaterials, and respondents’ knowledge about the possibilities of using mycelium. It comprised five questions:

- Question 1.1. Do you believe ecology plays a significant role in shaping contemporary architecture?
- Question 1.2. Do you incorporate biodegradable materials in your architectural or interior design projects?
- Question 1.3. If so, what specific biodegradable materials have you used in your designs?
- Question 1.4. Are you familiar with mycelium-based composites (MBCs) as a biomaterial?
- Question 1.5. Have you heard of using any MBC as a building material?

Section 2. Evaluation of the aesthetics of implementations utilizing mycelium on a five-point Likert scale. The second section contained photos of selected implementations and questions regarding their aesthetics. Three questions were posed for each of the presented examples:

- Question 2.1. Please evaluate the overall form of the small-scale architectural objects.
- Question 2.2. To what extent do you find the architectural details of these objects to be well-executed and precise?
- Question 2.3. In your opinion, do the designs exhibit a high degree of visual interest?

Section 3. Assessment of material acceptance level and personal impressions.

- Question. 3.1. How visually appealing do you find mycelium-based composites (MBCs)?
- Question. 3.2. How visually attractive or harmonious do you find MBCs?
- Question. 3.3. In your opinion, do MBCs offer creative possibilities for shaping small architectural forms?
- Question. 3.4. Would you consider MBCs in your professional design work?
- Question. 3.5. Would you consider MBCs in a design project for your personal use?
